# Supplementary material for: From protection of sacrificial self to critical turning points and growth: Redeployed nurses’ experiences on the frontline during the COVID-19 pandemic
Source: PLoS One. 2025 Aug 21;20(8):e0314830. doi: 10.1371/journal.pone.0314830 (PMC12370062; doi:10.1371/journal.pone.0314830)
Supplement: S1 Table — (DOCX) [file pone.0314830.s001.docx]

| **Super-ordinate Themes** | **Sub-ordinate Themes** | **Sub-themes** | **Quotation examples** |
| --- | --- | --- | --- |
| **Theme 1:**  **Protection of Sacrificial Self** | **Intense psychological Overwhelm** | Existential threat  Disrupted sense of time  Fear  Feeling angry guilty and despair  Moral distress and injury  Forced into making critical decisions  Disrupted sense of time  Flashbacks | ‘to the emergency department, that I was so planned in my head that if I had to fill the red form to do not resuscitate me … that just kind of ... broke me … we worked on the patient for 45 minutes ..., if I had to go through that stage …, I would just not want to be resuscitated’ (Claire 378-394)  ‘I needed to be prepared and this was serious, and I needed to just be responsible, so I rewrote my will. So, I knew back then or in March, I felt I was being asked to go into a very dangerous situation but I, I was very prepared to do it because I was a nurse’(Una 42-46)  “It was, it was worse than anyone ever anticipated, really. And it was trying to find staff, trying to find nurses, trying to find health care assistants, literally just trying to find them to come in” (Ruth 546-549)  ‘Because you have no events, you've no occasions to mark your time anymore … it's Ground Hog Day, … I really struggled to keep track of it…the days are horrendously slow, but the year has flown’ (Trish 355-361)  “I used to feel “when will this shift get finished?”, and I don't want my patients to die on my watch. It was literally like, to finish off this shift, push it off to someone, that I have handed them over safely, to the other nurse coming on, so those were the emotions that were that were going on in my head” (Claire 88-93)  ‘I would have thought, is there any escape ...? Like if there was a door … I would happily run out that door … it was the anger and expecting so much from you … and you were pulled left, right and centre.’ (Claire 99-104).  “a lot of tears were shed like, I'm coming home and then being at home because it wasn't something you could escape, you know, if you went, you're just consumed at work, you know, there were many days and shifts that you ‘I just didn't get a break because you were just under so much pressure and you were still busy, and then if you did come home and go through your phone to try and debrief to and just escape it, like your whole feed is just COVID related news.” (Emma 93-101)  When you listen back to this now you will hear how difficult, difficult, difficult it was. And even I suppose the memory of it now is … even fading a wee bit…I consoled myself by saying I did my very best. It just wasn't good enough…it was what I could do but it wasn’t good enough (Ruth 565-577)  ‘I took on everything, and if someone was deteriorating, it was because I wasn't doing a good enough job or if I had missed something…I felt like it would have been my fault … (Emma 309-313)  ‘I don't want people to go in double black bags … I feel like the dignity of the person at the time of their death, or even after they died, I'm not a grocery material to go in a plastic bag … I don't want to pack anymore bodies; I don't want to do that’ (Claire 900-905)  ‘You can just foresee all these things happening that might not actually ever happen, and there were plenty of things I thought would happen and…never did. So, I think I put myself through these horrible situations…twice.’ (Emma 296-302)  ‘When you listen back to this now you will hear how difficult, difficult, difficult it was. And even I suppose the memory of it now is … even fading a wee bit…I consoled myself by saying I did my very best. It just wasn't good enough…it was what I could do but it wasn’t good enough’ (Ruth 565-577)  ‘…there’s like butterflies in my stomach when I just go in through that door, because I'm re-living that moment every time…like, my God, this is where I saw the BIPAP being pulled’ (patient taken off ventilator) (Claire 708-716)  ‘One day I was leaving work and … you scrub your hands, and you scrub your hands, and you scrub your hand, and you put on your coat, and you go out into the air with your mask on, and somebody asked me what time is it and I looked at them and said “I'm so sorry, I'm too tired to tell you… yeah that’s a flashback’ ​(Una 812-825)  ‘I didn't realize that I would be crying ... at times I do let cry and ventilate … even for the simplest of things … I was crying, there was no reason for me to cry, but I would be in tears, I don't know why’ (Claire 560-566) |
|  | **Sacrificial Commitment** | Moral duty  Volunteering extra time  Duty to protect patients and family  Personal sacrifices | ‘I wasn't fighting the fact that I needed to go in and do this. I knew I needed to go in and do this, like I knew it was the right thing to do, so there was no doubt in my mind even if I got COVID.’ (Una 584-588)  ‘I sat with quite a lot of patients … so they wouldn't be on their own, which I wouldn’t have done before, but I made an effort … I'd stay on after shift…I used to be with them and just give them that little bit extra attention and care, just because they weren’t going to get it when, when they did die…’ (Emma 1377-1384)  ‘I had this emotional connection with my patients as well, and I was more important for their family members. I was someone's daughter; I was someone’s family member’ (Claire – Lines 680 - 683)  ‘I… had to go to City West for my isolation…because obviously there were quite a few adults in my house …. I just didn't have the room really, just to stay in one room.’ (Ruth 718-732)  ‘…you can pull yourself because you're like an elastic band and it can only be stretched so far’ (Ruth 904-906)  ‘…that's the way I felt because I was so sick at one stage … I said like I don’t want anything, I don’t want this life, and I wanted to just go home and to my mom, just to go to her lap and cry.’ (Claire 366-378) |
|  | **Emotional detachment of self to cope** | Potent use of metaphors  Disengaging from news and social media  Putting feelings on hold  Pausing to rest | ‘Well, you walk into a ward, you might not have been in there for three hours, you don't know what you're going to get, the person could be dead! There are bodies going out every single day. There are just all these people crowding in. It's like “Mash”… coming in on trollies, barely able to breathe, frightened, anxious. I mean, it was a war zone’ (Una 609-616)  ‘I just stayed away from news or anything medical based because it just, would either be a trigger for me or just would, just send me down a path that would get me back thinking about work’ (Emma 398-402)  ‘I can't let myself fully process the past year, because I don't know if I'm gonna fall apart or not, … am I gonna hate my job and never want to go back…if we start kind of questioning ourselves, am I OK to do this, we're not going to turn up’ (Trish 1113-1123)  ‘It was a very therapeutic time for me… I didn't have to think about anyone, only myself and … gradually… when I was getting better, I was able to read, I was able to go out for a walk …’ (Ruth 737-751) |
|  | **Hunkering Down** | Inducing a protective flow-type state  Focusing on the present moment | ‘You had to just make yourself concentrate on what you were doing, like, I kind of describe it like it's working in a hole, because my world was what was going on in front of my two eyes, and I did not have time for anything else around me …’ (Una 1004-1011)  “I did feel like a headless chicken I didn't know where to start or you know, but then the next day once I got in, I felt that I had some, there was some rhythm to work.” (Ruth 800-804) |
| **Theme 2:**  **The Fortifying Effect of Us** | **Abandonment enhances solidarity** | Feeling isolated and unsupported  Feeling abandoned and exploited  A developing them and us | ‘…to even see a face …, would have meant a lot to us…one visit in a year to the unit … really made us feel aware of where we are in the pecking order of importance…’ (Trish 958-966)  ‘I don't love nursing as much as I used to, and I would like…to maybe leave and pursue something else … I don't feel that my service was valued’ (Denise 1246-1253)  ‘I think a lot of us just felt like it was a kick in the teeth…we really just felt like a number…you know, redeployment was part of the gig, so “what do they have to complain about?” so yeah, it was just disheartening’ (Emma 1088-1093)  “we weren't really given much notice, we were phoned over the weekend and told “you’re going to this ward” on this week, and I said, “hold on now, we have to cancel due clinics and we have to do a few things”, so they, so “you can have Monday to do that and then you’re going” (Denise 74-79)  ‘… it's more difficult because we're with the patient all day, I mean the rest of them will do their treatment and … go, the doctor will come in and do their treatment and they'll go, the dietitian will come in and do her treatment and go, you know? So, we were the ones that are there all the time, yes, the constancy of it’ (Denise – Line 1362)  “Am I allowed to say that I would love to go slap people who say there is no such thing as COVID? … because they haven't seen what we have seen in that place, they were not there holding the hands of a person that was dying” (Claire 815-821) |
|  | **The universality of us** | Began to turn towards each other and merge  Felt a shared sense of meaning and purpose globally  Tributes a reminder of progress | “But from the lessons learned, I would (pause)… look after myself and look after my colleagues, that's all I can do … because that was one thing that was poorly done, nobody ever had come back to us even asking us how we felt” (Claire 905-916)  “and then on duty, it’s about having one another’s back, isn't it? You’re seeing that somebody else is busy on one particular side and maybe you help them out if you can or whatever, helping out I suppose, yeah, anticipating something for them, trying to facilitate breaks because you have to have a break too, you can't stay on duty without a break” (Ruth 349-345)  “I think there's some kind of comfort there as well to know that it's happened all around the world and … most nurses have gone through it all together, so there is some kind of unity yeah, and like solidarity’ (Emma 517-521)  “we absolutely bonded; when somebody was sick, the WhatsApps were flying, you know that the support and they were near strangers at that time (Yeah), and now I know nurses do this; you work, you know, you work one shift with somebody,”(Trish 198-203)  ‘… you're just tighter with your colleagues because you had to get through this together’ (Una 432-434)  ‘We kind of like poured our hearts out and we were able to…vent our feelings with each other, to share our emotions’ (Claire 1013-1015)  ‘There was like an open area where there were pictures of us, socially distanced together; so those pictures went up on the positivity board, something to look back and say “OK, fine we have survived this”’ (Claire 200-204) |
|  | **Growing self-belief** | Growing self-efficacy  Feeling better able to cope  More open mindset  Feeling less of a victim  Rebuilding internal belief systems | “I'll forget all this potentially … because you go back to normal, and you just carry on. It is an appreciation, more self-awareness … a lot of time for introspection and … wondering why do I think what I think?” (Trish 829-835)  “I know what to do to kind of de-stress that I know that I need, that I need to eat something like, I always do this thing with my child. It's called H.A.L.T., you might have heard of it; Are you hungry, angry, lonely or tired, right? Hungry, angry, lonely or tired? So, if I find myself getting annoyed, I can say alright, am I hungry, am I angry and am I lonely or am I tired, so what's going on for me here. So, I'd be good at checking in with myself” (Denise 1016-1025)  “once you get over the initial stress of the redeployment and being thrown into a new environment and having to do things you haven't done for a long time, you know and, once you kind of, you think, “Oh yeah, I remember this now” and the knowledge starts to come back and you start to get to know the environment”(Denise 1052-1059)  “I don't want another wave coming, but if it does come to it, I would be willingly and happy going there” (Claire 935-937)  ‘I've changed because I'm completely exhausted and apathetic and burnt out. I don't know how to start again in my own job a second time … however, me being me, I signed myself up for a … course in the area I work in, to help me find the enthusiasm again for work because, you know, I actually love the job I'm in’ (Una 707-716) |
| **Theme 3: Critical turning points and growth** | **Acceptance and Adjusting Expectations** | Introspection  Enabled significant changes  Gratitude  Progress and growth  Accept the new way of working | ‘So, anger was the first thing, like there was no reason to be angry with everyone, I was angry within myself like, that I wasn't able to get on top of doing my things, which I would have normally done on a day-to-day basis looking after my patients’ (Claire 64-68)  ‘I had one particularly bad shift … I remember getting up the next day … and painting surfing waves … and then there's this tiny little figure in a life jacket down at bottom and that's me. It ended up that really helped you know because … you know, I'm OK, I will survive this so’ (Denise 474-481)  ‘“Thank you, our heroes” and I saw it and I just burst out crying like you know, that child, he doesn't know that, but…it did make a massive difference’ (Una 829-834)  ‘I had feelings that the care delivered to the patients was sub optimal definitely, some of the time, not all of the time, and I had to reconcile that with myself with “I did my best, I did my very best’ (Ruth 766-769)  ‘but then when I was at work trying to actually cope with it … I just started to have the mindset of “you control the controllables”, you know?’ (Emma 289-292) |
|  | **Revitalised Nurse Self** | Positive self-talk  Finding voice  Reaffirming their contribution  Reconciliation | “I just had to talk to myself and tell myself I could do it and just get on with it and stay calm and stay focused” (Una 106-108)  “Well as a nurse, I think I’ve found my voice: if there is a sign of deterioration or anything … definitely I feel more confident in myself. Definitely more confident in my ability and my experience and my knowledge” (Emma 877-892)  ‘I'm proud of myself for going in and having put my shoulder to the wheel and, you know, pushed with the rest of my colleagues, em, I didn't run away’ (Una 653-657)  ‘I'm not forcing myself to do anything…whatever happens happens…just kind of finding peace with that as well and just trying to switch off’ (Trish – Lines 895 – 899)  ‘I might have not done everything, but I was there for someone, and I've helped out, I have touched someone’s life. I was able to offer that service, and I feel so good about it myself, that I have chosen this profession’ (Claire 1097-1101)  “I suppose there's always going to be the ones that stay with you, you probably know that, there's ones you kind of bond with or you like you know, or you get on with, you feel some affinity towards and you always kind of wish you know, want them to do, you want them to, you know, you want everyone to do well, but you might want them to do well in a special way” (Denise 976-983)  ‘…so many times, I've taken my life for granted, and I reflected it so much to the patients like. I am fine, I am healthy … Imagine someone who's so very sick with all other underlying conditions, how hard it must be for them in that hospital bed.’ (Claire 579-587) |
|  | **Renewed self** | Feeling pride and gratitude  Taking comfort in family friends and the ordinary things in life  Taking comfort in nature  The interviews fostered meaning and purpose – served as debriefing | “how you are with people is really a lot of how you are with yourself you know, so if you're OK with yourself, and I think that's a big thing with COVID. I am OK with myself so, I will always be OK with the patient really”(Denise 1002-1006)  But it was really my family, like coming home to my husband now and he having the dinner cooked and getting into a nice lovely hot shower and then getting into my pyjamas and then getting a hug from my child’ (Denise 507-511)  ‘The sea is very calming, it's just like meditation, it's full of life, it's so positive, … it’s the simple things that I enjoy …, baking in the kitchen, family and friends, a warm fire in the evening, a home cooked meal, barbecues in the garden, dogs, animals, cats’ (Una 740-752)  ‘You touched on every aspect of it and it's great, like, I feel this itself as a debriefing session, treatment, just kind of talk about it and thank you. Thank you for doing such a study’ (Claire 1109-1112) |
