# Supplementary figures and images for: From protection of sacrificial self to critical turning points and growth: Redeployed nurses’ experiences on the frontline during the COVID-19 pandemic
Source: PLoS One. 2025 Aug 21;20(8):e0314830. doi: 10.1371/journal.pone.0314830 (PMC12370062; doi:10.1371/journal.pone.0314830)

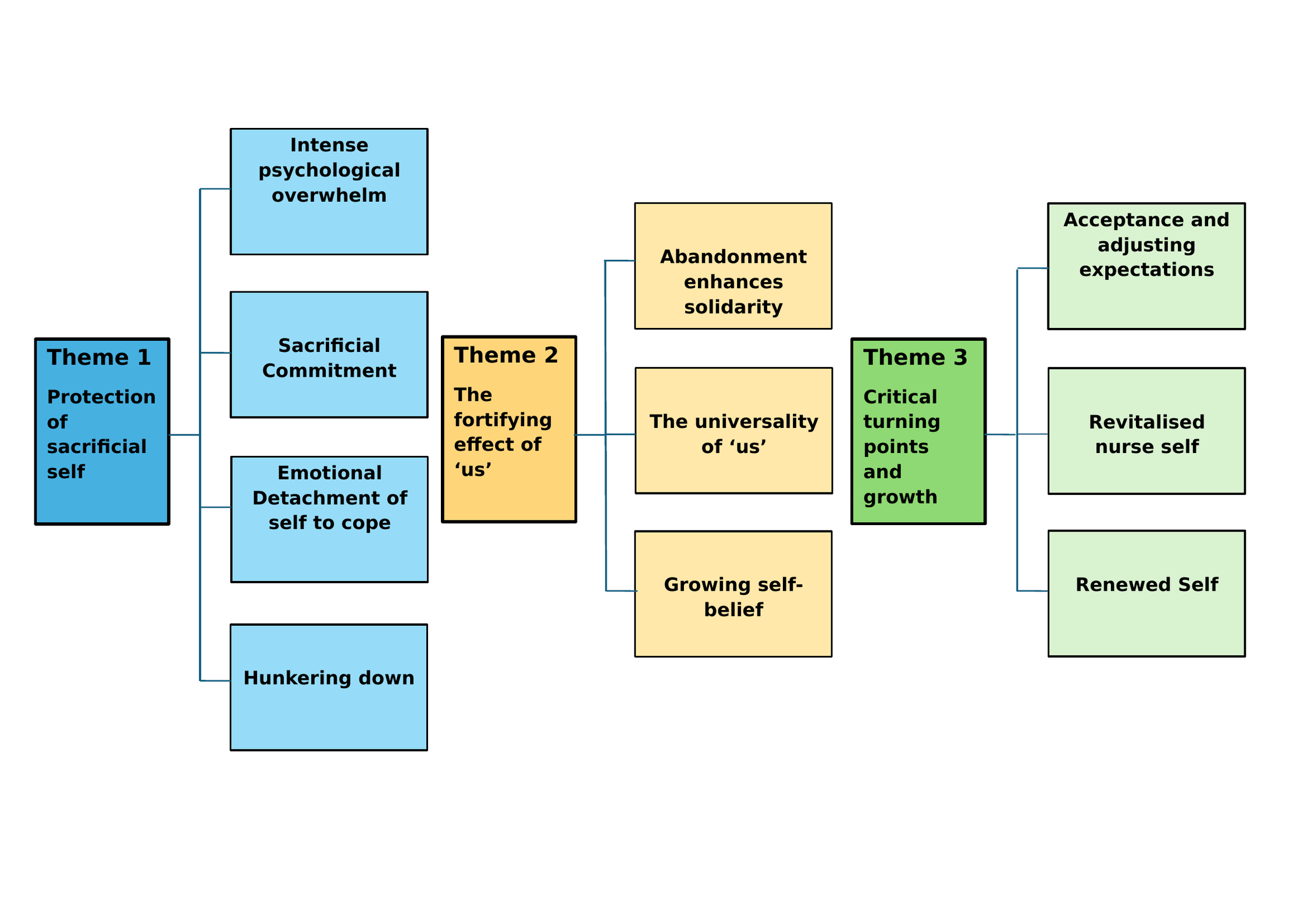

Supplement: S1 Fig — (TIF) [file pone.0314830.s002.tif]
